# Interpretable Machine Learning Models for Predicting Critical Outcomes in Patients with Suspected Urinary Tract Infection with Positive Urine Culture

**DOI:** 10.3390/diagnostics14171974

**Published:** 2024-09-06

**Authors:** Chieh-Ching Yen, Cheng-Yu Ma, Yi-Chun Tsai

**Affiliations:** 1 Department of Emergency Medicine, Chang Gung Memorial Hospital, Linkou Branch, Taoyuan 33305, Taiwan; 2 Department of Emergency Medicine, New Taipei Municipal Tucheng Hospital, New Taipei City 23652, Taiwan; 3 Institute of Emergency and Critical Care Medicine, National Yang Ming Chiao Tung University, Taipei 30010, Taiwan; 4 Department of Artificial Intelligence, Chang Gung University, Taoyuan 33302, Taiwan; cyma@cgu.edu.tw; 5 Artificial Intelligence Research Center, Chang Gung University, Taoyuan 33305, Taiwan; 6 Division of Rheumatology, Allergy and Immunology, Chang Gung Memorial Hospital, Taoyuan 33305, Taiwan; 7 Department of Nursing, Chang Gung University of Science and Technology, Taoyuan 33303, Taiwan; ycn.tsai@gmail.com

**Keywords:** machine learning, urinary tract infection, predictive model

## Abstract

(1) Background: Urinary tract infection (UTI) is a leading cause of emergency department visits and hospital admissions. Despite many studies identifying UTI-related risk factors for bacteremia or sepsis, a significant gap remains in developing predictive models for in-hospital mortality or the necessity for emergent intensive care unit admission in the emergency department. This study aimed to construct interpretable machine learning models capable of identifying patients at high risk for critical outcomes. (2) Methods: This was a retrospective study of adult patients with urinary tract infection (UTI), extracted from the Medical Information Mart for Intensive Care IV Emergency Department (MIMIC-IV-ED) database. The critical outcome is defined as either in-hospital mortality or transfer to an intensive care unit within 12 h. ED visits were randomly partitioned into a 70%/30% split for training and validation. The extreme gradient boosting (XGBoost), random forest (RF), and support vector machine (SVM) algorithms were constructed using variables selected from the stepwise logistic regression model. The XGBoost model was then compared to the traditional model and clinical decision rules (CDRs) on the validation data using the area under the curve (AUC). (3) Results: There were 3622 visits among 3235 unique patients diagnosed with UTI. Of the 2535 patients in the training group, 836 (33%) experienced critical outcomes, and of the 1087 patients in the validation group, 358 (32.9%) did. The AUCs for different machine learning models were as follows: XGBoost, 0.833; RF, 0.814; and SVM, 0.799. The XGBoost model performed better than others. (4) Conclusions: Machine learning models outperformed existing traditional CDRs for predicting critical outcomes of ED patients with UTI. Future research should prospectively evaluate the effectiveness of this approach and integrate it into clinical practice.

## 1. Introduction

Urinary tract infection (UTI) is a prevalent condition affecting individuals of all ages, with particular significance among elderly patients. It is a frequent cause of primary healthcare visits and hospital admissions, accounting for up to one-quarter of emergency department visits, especially in certain high-risk groups [1]. It poses a significant healthcare burden and can lead to severe conditions such as urosepsis, which accounts for a substantial portion of sepsis cases, leading to increased morbidity and mortality, especially when complicated by underlying urinary tract or systemic diseases such as anatomical abnormalities, diabetes mellitus, steroid use, or chemotherapy [2,3]. Despite the existence of numerous studies aimed at identifying risk factors for bacteremia or sepsis in the context of UTI [4,5,6,7,8], there remains a significant gap in the research, particularly concerning the development of predictive models for critical outcomes such as in-hospital mortality or the necessity for emergent intensive care unit (ICU) admission in the emergency department (ED). Therefore, identifying patients at high risk of rapid deterioration through risk assessment models can help in timely treatment and allocating ED resources.

Machine learning offers a powerful tool for analyzing complex clinical datasets. Its capability to uncover patterns within extensive datasets positions machine learning as a critical asset in healthcare research, providing insights that enhance patient care [9]. However, the opaque nature of machine learning algorithms—often referred to as “black box” models—presents challenges in clinical adoption due to the difficulty in understanding their decision-making processes [10]. Recent advancements in interpretable algorithms are addressing these challenges, improving the transparency and trustworthiness of machine learning models in healthcare settings [11,12]. These algorithms are increasingly used in the field of infectious diseases, such as prognosticating COVID-19 outcomes and diagnosing malaria [13,14].

This study aimed to develop machine learning models to predict critical outcomes in patients with suspected UTI with positive urine culture in the ED and used an interpretable machine learning algorithm to interpret the decision-making processes.

## 2. Materials and Methods

### 2.1. Data Source and Population

This study utilized data from the MIMIC IV Emergency Department (MIMIC-IV-ED) database [15,16], a publicly available and anonymized clinical database. The database encompasses over 400,000 ED visit episodes from a wide range of centers, specifically Beth Israel Deaconess Medical Center in Boston, MA, USA, spanning from 2011 to 2019. The first author was granted access to these comprehensive resources after completing the necessary training course from the Collaborative Institutional Training Initiative, receiving Collaborative Institutional Training Initiative certificates (No. 59843370). Since the MIMIC database is publicly available and anonymized, ethical committee approval was not required for this study. This study adhered to the Transparent Reporting of a multivariable prediction model for Individual Prognosis or Diagnosis Statement (TRIPODS) [17]. In this study, we considered all patients over 18 years old with suspected UTI associated with cystitis or pyelonephritis who were admitted to the ward or ICU from the ED for inclusion. We extracted patient data from the MIMIC-IV database using ICD-9 and ICD-10 codes for those diagnosed with UTI, which were previously validated in a large electronic health record (EHR) system [18]. To confirm the UTI diagnosis, we relied on positive urine culture results, excluding cases where urine cultures did not isolate any microorganisms.

### 2.2. Variable Extraction and Data Preprocessing

The hadm_id identifier of UTI patients was used to extract the following information: age, gender, race, insurance status, arrival method, emergency severity index (ESI), vital signs at triage, comorbidities, and first laboratory examination results. Comorbidities included hypertension, diabetes, prior myocardial infarction, congestive heart failure, prior stroke, liver disease, chronic kidney disease, malignancy, and Charlson Comorbidity Index calculated from previous parameters. The first laboratory test results after admission included levels of white blood cells (WBCs), neutrophils, monocytes, lymphocytes, hemoglobin, mean corpuscular volume (MCV), red cell distribution width (RDW), platelet count, prothrombin time, activated partial thromboplastin time (aPTT), sodium, potassium, aspartate aminotransferase (AST), Alanine Aminotransferase (ALT), total bilirubin, glucose, blood urea nitrogen (BUN), creatinine, albumin, calcium, magnesium, chloride, lactate, C-reactive protein, blood pH, bicarbonate, urinary WBCs, urinary red blood cells, urine pH, and the microorganism of urine culture. The primary endpoint of this study was the critical outcome during hospitalization. The critical outcome is defined as either in-hospital mortality or transfer to an ICU within 12 h.

**Figure 1:**
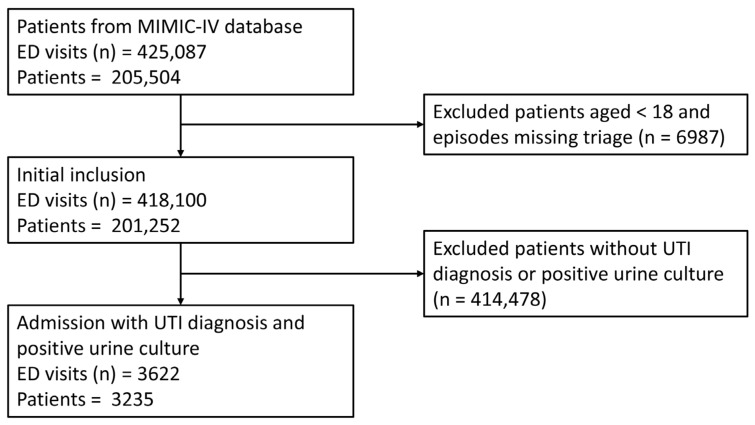
Flow diagram.

The data preprocessing steps and patient inclusion process are depicted in Figure 1. Raw EHR data are not suitable for direct use in model construction due to the presence of missing values, outliers, duplicates, and incorrect entries, which can result from system errors. To mitigate these issues, we implemented several procedures. For vital signs and laboratory tests, any value falling outside the plausible physiological range, as determined by domain expertise (e.g., a heart rate below zero), was considered an outlier and treated as missing. This approach to outlier detection aligns with the methodology utilized in MIMIC-EXTRACT [19]. Missing values were imputed using multivariate imputation by chained equations [20]. Multiple imputation involves two stages: first, generating replacement values for missing data and repeating this process multiple times, resulting in several datasets with replaced missing information; and second, analyzing these imputed datasets and combining the results. Creating multiple imputations, rather than a single imputation, accounts for the statistical uncertainty inherent in the imputation process. The final dataset was constructed following the rules established by Xie et al. [21].

### 2.3. Model Construction and Comparison

Our primary aim was to evaluate the predictive accuracy of big data-driven, machine learning approaches compared to traditional analytical models and previously developed clinical decision rules (CDRs). We developed four models using extreme gradient boosting (XGBoost), random forest (RF), support vector machine (SVM), and a multivariable generalized estimating equation (GEE) logistic regression model. The latter, adjusted for clustering in patients with multiple visits, represented the traditional approach, while XGBoost, RF, and SVM exemplified newer machine learning methods. A detailed description of the machine learning algorithm is presented in Supplementary Table S1.

Each of these models was trained and tested using a data split of 70% for training and 30% for validation. We fine-tuned the hyperparameters in the machine learning models using a randomized search strategy with five-fold cross-validation. Recognizing the high cost of false negatives in acute settings, we implemented a rule-out strategy that adjusted the classification threshold to 0.2, meaning a model predicts a positive result if its output probability exceeds this threshold, and negative otherwise.

The performance of these models was compared with established CDRs, including the Modified Early Warning Score (MEWS) [22], National Early Warning Score (NEWS) [23], and Rapid Emergency Medicine Score (REMS) [24]. It is important to note that the MIMIC-IV-ED dataset lacks neurological variables, such as those measured by the Glasgow Coma Scale (GCS), which could result in incomplete scores. We assessed model performance using various metrics: area under the curve (AUC) of the receiver operating characteristic (ROC) curve, accuracy, no-information rate, balanced accuracy, kappa, sensitivity, specificity, precision, and F1 scores. We employed DALEX (Descriptive mAchine Learning EXplanations), a tool in machine learning, to enhance the interpretability of the final model [25]. Its model-agnostic nature enables compatibility with any machine learning model. It offers both global- and instance-level explanations, providing insights into overall model behavior and individual predictions. This tool facilitates an understanding of the importance of variables within the model, their correlation with clinical outcomes, and the extent to which each variable contributes to individual predictions, thereby enhancing our understanding of the model’s decision-making process. We conducted subgroup analyses by stratifying patients based on the presence of fever at triage and systolic blood pressure of ≥100 mmHg or <100 mmHg. Additionally, we conducted a sensitivity analysis to develop new machine learning models for predicting in-hospital mortality, utilizing the same variables selected for models aimed at critical outcomes. We evaluated their performance and compared these models with the GEE logistic regression model and traditional CDRs.

### 2.4. Statistical Analysis

We calculated descriptive statistics as numbers and percentages for categorical data, while continuous variables were reported as the median and interquartile range. We compared the clinical characteristics of the training and validation groups using the independent Student’s *t*-test for normally distributed data, or the Wilcoxon rank-sum test for non-normally distributed data, as appropriate. Differences in categorical variables were compared using Pearson’s chi-square or Fisher’s exact test. A two-sided *p* value < 0.05 was regarded as statistically significant. Variables exhibiting a missing data proportion of less than 50% were selected as potential predictors. The final selection of predictors in the subsequent models was made using a stepwise logistic regression approach. The Akaike Information Criterion (AIC) served as the guiding metric for this stepwise variable selection process [26]. At each step of the process, we calculated the AIC, employing the forward selection and backward elimination of predictor variables. This iterative procedure continued until the addition or removal of variables ceased to enhance the AIC, leading us to a model with the minimal AIC value. We evaluated the performance of each model on the validation cohort using ROC analyses and utilized 2000 bootstrapped samples to determine the 95% confidence intervals and select the final model. We used R version 4.1.2 for descriptive analyses and the GEE regression model, while the other machine learning models were constructed using Python (version 3.11.5).

## 3. Results

### 3.1. Patient Characteristics of the Training Group and Validation Group

A total of 3622 ED visits among 3235 unique patients were enrolled in the cohort of this study, with 2535 visits in the training group and 1087 visits in the validation group (Figure 1). Males accounted for 34.4% and 34.3% of the UTI patients in the training and validation groups, respectively, while the median age [interquartile range] was 72.0 [60.0–81.0] and 71.0 [59.5–81.0] years. In the training group, 836 patients (33%) experienced critical outcomes, including 766 (30.2%) ICU admissions and 153 (6%) in-hospital mortalities. Meanwhile, the validation group had 358 patients (32.9%) with critical outcomes, comprising 329 (30.3%) ICU admissions and 67 (6.2%) in-hospital mortalities. Patient demographic and clinical characteristics of the training and validation groups are presented in Table 1. The frequency of missing data is described in Supplementary Figure S1.

### 3.2. Variable Selection and Model Performance Comparisons

**Figure 2:**
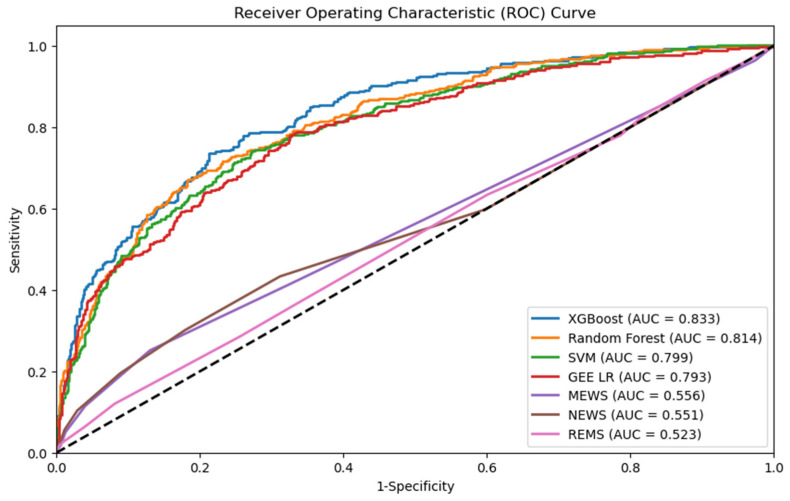
Comparison of AUC of four models to traditional prediction tools for predicting critical outcomes in patients with UTI. AUC: area under the curve; UTI: urinary tract infection.

Through stepwise logistic regression, we finally selected 20 variables, which were ESI, arrival by ambulance, mean arterial pressure (MAP), WBCs, hemoglobin, MCV, platelet count, aPTT, sodium, BUN, creatinine, AST, ALT, calcium, magnesium, chloride, glucose, albumin, lactate, and bicarbonate (Table 2). XGBoost, RF, and SVM algorithms were used to construct models. The five-fold cross-validated random search strategy resulted in the finalization of the hyperparameters for XGBoost as column sample by tree = 0.7, minimum loss reduction required to make a further partition on a leaf node of the tree = 0.0, learning rate = 0.09, maximum number of levels in each decision tree = 7, minimum sum of instance weight (hessian) needed in a child = 2, number of gradient-boosted trees = 343, L1 regularization term on weights = 0.01, L2 regularization term on weights = 100, and subsample ratio of the training instances = 0.6; for RF as maximum number of levels in each decision tree = None, minimum number of samples required to split an internal node = 4, number of trees in the forest = 53, and number of features to consider when looking for the best split = 1; and for SVM as penalty parameter C of the error term = 3.78, kernel type = radial basis function, and degree of the polynomial kernel function = 3. We used AUC, accuracy, no-information rate, balanced accuracy, kappa, sensitivity, specificity, precision, and F1 scores to comprehensively evaluate the model’s performance. XGBoost had the largest AUC (0.833, 95% CI: 0.806–0.857), followed by RF (AUC: 0.814, 95% CI: 0.785–0.839), SVM (AUC: 0.799, 95% CI: 0.771–0.826), and GEE logistic regression model (AUC: 0.793, 95% CI: 0.764–0.820). The performance of traditional CDRs was inferior to that of machine learning models, with the AUC for MEWS, NEWS, and REMS being 0.556, 0.551, and 0.523, respectively (Figure 2). We selected XGBoost as the final model since the AUC, accuracy, balanced accuracy, kappa, precision, and F1 score of XGBoost were higher than those of RF, SVM, and GEE logistic regression models, as shown in Table 3 and Supplementary Table S2. Variance inflation factor values in the logistic regression model are presented in Supplementary Table S3.

Subgroup analyses showed that the XGBoost model maintained acceptable performance in patients with fever at triage (AUC: 0.758) and in those with systolic blood pressure of either ≥100 mmHg (AUC: 0.825) or <100 mmHg (AUC: 0.870) (Supplementary Table S4). The sensitivity analysis showed that when using the same variables, XGBoost consistently outperformed other models and traditional CDRs in predicting in-hospital mortality. Detailed performance metrics for each model are presented in Supplementary Table S5 and Supplementary Figure S2.

### 3.3. Interpretation of the Machine Learning Model

**Figure 3:**
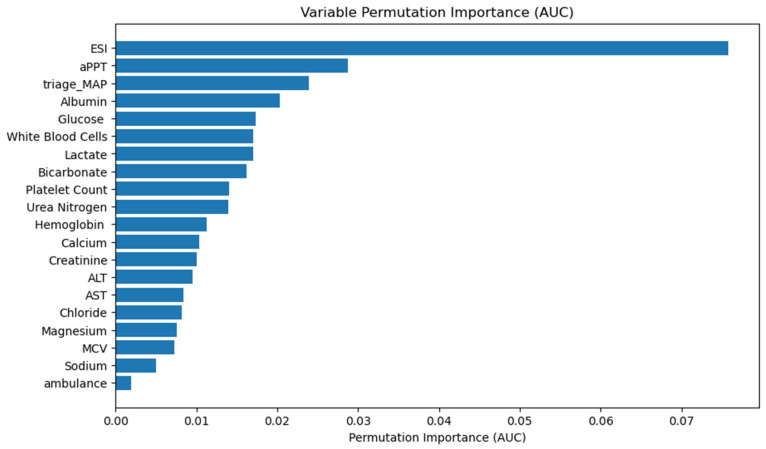
Variable permutation importance is derived from the XGBoost model. The X-axis quantifies the decrease in AUC following the random permutation of a variable, compared to the original AUC. A greater decrease indicates higher importance of the variable to the model’s performance. AUC: area under the curve; ESI: emergency severity index; aPTT: activated partial thromboplastin time; MAP: mean arterial pressure; ALT: alanine aminotransferase; AST: aspartate aminotransferase; MCV: mean corpuscular volume.

Figure 3 shows the permutation importance of the independent variables ranked in descending order in the final XGBoost model. The top five most important features in predicting critical outcomes were the ESI, aPTT, triage MAP, albumin, and glucose. We also described the effect (positive or negative) of clinical characteristics on the model as shown in Supplementary Figures S3–S6. Variables associated with increased risk of critical outcomes were higher AST, ALT, glucose, BUN, and WBCs, while a lower ESI level, calcium, and platelet count were associated with an increased risk of critical outcomes. In addition, arrivals by ambulance were also helpful in predicting critical outcomes. We also found that bicarbonate, albumin, chloride, aPTT, and lactate were associated with an increased risk of critical outcomes in a U-shaped curve.

## 4. Discussion

In this study, based on data from the MIMIC-IV database, we developed four predictive models—XGBoost, RF, SVM, and a traditional GEE logistic regression model—to predict critical outcomes in patients with suspected UTI in the ED. To the best of our knowledge, this study represents the largest effort to date in constructing machine learning models for predicting outcomes in UTI patients in the ED. Notably, the XGBoost model exhibited superior performance, emphasizing the efficacy of machine learning approaches over traditional models and CDRs in predicting critical patient outcomes. Through a stepwise approach and an extensive variable importance analysis, we identified twenty key variables, including ESI, aPTT, MAP, albumin, glucose, lactate, WBCs, bicarbonate, BUN, platelet count, hemoglobin, creatinine, ALT, calcium, AST, chloride, magnesium, MCV, sodium, and arrival by ambulance, which influenced the model’s predictions. Moreover, the utilization of the DALEX tool for interpretability underscores our commitment to transparency and understanding in machine learning applications in healthcare, offering a detailed view of how individual predictors contribute to the model’s decision-making process.

In recent years, machine learning techniques have become a mainstream approach extensively used in the field of medical diagnoses. Their performance often exceeds that of traditional prediction tools, which typically rely on ordinary least squares regression techniques known for their straightforward interpretability through coefficients. However, the capability of machine learning to capture nonlinear interactions between features introduces complexities in understanding how input features relate to output predictions. These nonlinear algorithms, often labeled as ‘black box’ structures, are opaque, non-intuitive, and challenging for individuals to comprehend the decision-making process. This opacity limits the clinical application of these advanced models. The critical need for interpretability in all machine learning-based decision-making algorithms is highlighted by the United States Government’s Blueprint for an AI Bill of Rights, which emphasizes “Notice and Explanation” as a fundamental principle for ML-based prediction models [27]. Furthermore, the U.S. Food and Drug Administration (FDA) guidelines for clinical decision support systems (CDSSs) emphasized the importance of providing the basis of predictions [28]. In our study, we demonstrate how the interpretability and explainability of our machine learning models are informed by the application of so-called “model agnostic” methods at both the global and individual levels. Global interpretations aim to uncover the average decision-making processes of models at a cohort level, whereas individual interpretations delve into the behaviors of models for specific predictions. Our machine learning models play a crucial role in optimizing the allocation of healthcare resources. They ensure that high-risk patients are promptly and effectively identified and treated, thereby enabling the development of personalized care protocols.

**Figure 4:**
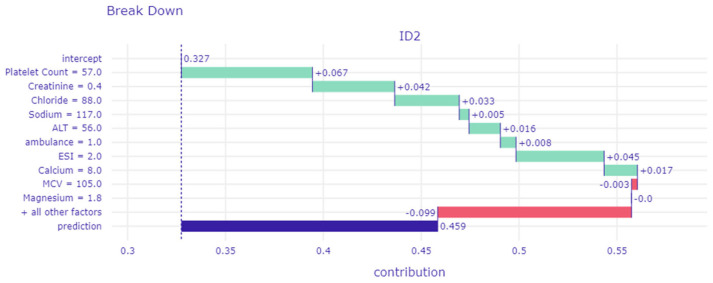
The break-down profile offers a detailed visualization of how individual variables contribute to a model’s prediction for a specific observation. It decomposes the prediction into components, each representing the contribution of a single variable to the overall prediction.

To further decompose the model’s prediction into contributions that can be attributed to different explanatory variables in individual patients, we randomly selected two patients from the validation cohort for a detailed analysis. Using interpretable algorithms, we can determine how an explanatory variable influences the prediction of outcomes, either by increasing or decreasing its probability. We presented one of these patients in the main text, while the other is detailed in Supplementary Figure S7. This patient, a 54-year-old female, presented with general weakness and arrived by ambulance. At the initial ED presentation, her ESI level was two, and the triage MAP was 79 mmHg. Laboratory data showed a hemoglobin level of 8.7 g/dL, MCV at 105 fL, platelet count at 57 K/uL, and white blood cell count at 5 K/uL. Her albumin level was at 3.4 g/dL, serum creatinine at 0.4 mg/dL, aPTT at 62.1 s, glucose at 91 mg/dL, sodium at 117 mmol/L, urea nitrogen at 19 mg/dL, and total bilirubin at 0.7 mg/dL. ALT and AST levels were 56 U/L and 57 U/L, respectively. Additional markers indicated bicarbonate at 23 mmol/L, magnesium at 1.8 mmol/L, calcium at 8.0 mg/dL, chloride at 88 mmol/L, and lactate at 1.76 mmol/L (Figure 4). The XGBoost model predicted a 45.9% risk of critical outcomes based on the patient’s initial ED presentation clinical characteristics, with platelet count, creatinine, ESI, chloride, and aPTT identified as the top five contributors to the increased risk of critical outcomes, while WBCs notably reduced the model’s prediction the most. The predicted outcome was that the patient would experience critical outcomes based on the threshold of 0.2, which was consistent with the actual outcome (true positive).

Our study revealed that lower ESI levels, signaling higher urgency, correlated with an increased risk of adverse events in UTI patients, notably ranking as the most critical factor in permutation importance. This correlation is likely due to ESI triage relying on clinical judgment through visual assessment by experienced personnel, allowing for the early identification of critically ill patients without the need for vital signs or laboratory parameters. Moreover, ESI evaluates patients for signs of confusion, distress, or severe pain, demonstrating high sensitivity and specificity in predicting in-hospital mortality [29]. Previous research supports the importance of ESI in patients with sepsis [30], but our findings specifically highlight its predictive value for UTI patients.

Our study illustrated that aPTT, lactate, bicarbonate, chloride, and albumin were associated with an increased risk of critical outcomes in a U-shaped curve. The previous literature has shown that septic patients with worsening hypercoagulability or impaired coagulation in ICUs have a higher risk of death [31]. Animal model studies have indicated that blocking distinct elements of the hemostasis process, such as factor VII and tissue factors, can increase organ failure or mortality [32,33], thereby explaining the correlation of aPTT and critical outcomes in UTI patients in our study. Extensive research has highlighted that higher lactate levels serve as a prognostic indicator for adverse outcomes in critically ill patients [34,35]. Our study introduces a nuanced perspective by identifying a U-shaped correlation, suggesting that risks may also be associated with abnormally low lactate levels. The underlying mechanism remains unclear, indicating the need for further investigation in this area. Serum bicarbonate has shown a U-shaped association with all-cause mortality in patients with chronic kidney disease [36]. Recently, one study demonstrated that base deficit levels exhibit a U-shaped relationship with 28-day mortality in sepsis patients, with both metabolic acidosis and alkalosis playing roles in patient prognoses [37]. These studies confirm our findings that patients with UTI exhibit a U-shaped correlation between bicarbonate and critical outcomes. Both low and high levels of chloride are associated with higher mortality rates in specific ICU populations [38], and this association was also found in UTI in our study. Studies indicate that high chloride levels may contribute not only to metabolic acidosis but also to greater hemodynamic instability [39]. We also discovered a U-shaped correlation between serum albumin levels and critical outcomes. Both hypoalbuminemia and hyperalbuminemia can indicate underlying health conditions or the severity of the disease. For instance, hypoalbuminemia may result from liver dysfunction, kidney disease, or gastrointestinal losses, whereas hyperalbuminemia could signal dehydration or, less commonly, overproduction in certain disorders [40]. This correlation has also been observed in critically ill children, indicating a risk of mortality, and in hospitalized patients, showing a risk of acute kidney injury [40,41].

This study has several limitations. First, although the dataset used in this study is extensive, enrolling over 3000 patients, it is derived from a single center. Different hospitals may have varying patient demographics, treatment protocols, and healthcare delivery systems, which could influence the predictive accuracy of machine learning models developed in this study. This limitation raises concerns about the generalizability of the findings to other healthcare settings, and the results obtained here may not be directly applicable or may require adjustment to be effective in different institutional contexts. Second, this study’s reliance on structured data from the EHR system means that potentially valuable unstructured data, such as physician notes, imaging reports, and patient narratives, were not included in the analysis. Additionally, reasons for deciding to admit patients to the ICU were not available from the EHR system. Unstructured data can offer rich, contextual insights that are often important for accurate diagnoses and prediction. The absence of this data type may limit the model’s ability to capture the full spectrum of patient information. Third, the lack of GCS scores in the MIMIC-IV-ED dataset poses a challenge to accurately predicting critical outcomes using established CDRs such as the MEWS, NEWS, and REMS. These CDRs often consider neurological status as a critical component. Treating missing neurological variables as normal, although a standard practice in clinical risk scores [35,42], may lead to inaccuracies in the predictive performance of these scores. Fourth, as a retrospective study, individual healthcare providers’ predictions of critical outcomes are unavailable, which would have served as a valuable comparator. This gap means that we could not assess the machine learning model’s performance against the clinical judgment of healthcare professionals, often considered the gold standard in medical decision-making. Such a comparison could have provided insights into the model’s relative strengths and areas for improvement. Fifth, our primary endpoints were composite outcomes consisting of in-hospital mortality or transfer to an ICU within 12 h, rather than predicting only in-hospital mortality, which is a more commonly used endpoint in prognostic predictive models. The main reason for this choice is that in-hospital mortality alone accounts for approximately 6% of all patients, leading to data imbalance during model building. This choice is supported by our sensitivity analysis, which shows that the performance of models predicting in-hospital mortality is slightly inferior to those predicting critical outcomes. Sixth, it is important to acknowledge the potential impact of multicollinearity among the variables in the logistic regression model. Some variance inflation factor values in the model were close to the threshold of five, indicating that multicollinearity could affect the stability and interpretability of the coefficients. This may reduce the precision of the estimated effects of individual predictors, making the model’s interpretation more challenging. Seventh, the inherent uncertainty in diagnosing UTI based solely on positive urine culture results presents a significant limitation. Although we used ICD codes and positive urine culture to define UTI cases, these criteria do not definitively confirm the presence of UTI, especially in patients who may have other sources of infection [43,44]. Therefore, our model predicts outcomes in patients with positive urine cultures rather than in those with a confirmed UTI diagnosis. Finally, this study conducted only internal validation without external validation. While internal validation helps ensure the model’s reliability within the same dataset, external validation is crucial for testing the model’s applicability to different patient populations and healthcare settings. The absence of external validation limits our ability to confirm the model’s generalizability and robustness across diverse clinical environments. Despite these limitations, this study lays the groundwork for integrating the machine learning model into our hospital’s operational framework to assess patients’ risk of critical outcomes in real time. We plan to conduct a prospective study to validate the model’s performance in a real-world setting. This step is important for translating the model from research to practice, ensuring that it can effectively aid in clinical decision-making and enhance patient care.

## 5. Conclusions

This study developed and validated interpretable machine learning models to predict critical outcomes in patients with UTI in the ED. Our findings highlight the potential of machine learning in enhancing patient care through the early identification of high-risk patients. The future integration of this model into clinical practice, coupled with real-world validation, is needed.

## Figures and Tables

**Table 1:** Demographics, clinical characteristics, laboratory results, and outcomes between training and validation cohort.

Variable	Training Cohort (*n* = 2535)	Validation Cohort (*n* = 1087)	*p* Value
Age (years), median [IQR]	72.0 [60.0–81.0]	71.0 [59.5–81.0]	0.408
Sex (male), *n* (%)	871 (34.4)	373 (34.3)	1.000
Race, *n* (%)			
Asian	81 (3.2)	27 (2.5)	0.326
Black	361 (14.2)	181 (16.7)	
Hispanic	108 (4.3)	46 (4.2)	
Other	192 (7.6)	77 (7.1)	
White	1793 (70.7)	756 (69.5)	
Insurance, *n* (%)			0.226
Medicaid	142 (5.6)	60 (5.5)	
Medicare	1468 (57.9)	662 (60.9)	
Other	925 (36.5)	365 (33.6)	
Arrival by ambulance, *n* (%)	1689 (66.6)	736 (67.7)	0.551
Emergency severity index, *n* (%)			0.328
Level 1	452 (17.8)	195 (17.9)	
Level 2	1307 (51.6)	563 (51.8)	
Level 3	768 (30.3)	329 (30.3)	
Level 4	8 (0.3)	0 (0.0)	
Vital signs at triage, median [IQR]			
Body temperature (°C)	36.7 [36.4–36.9]	36.7 [36.4–36.9]	0.499
Fever ^†^	170 (6.7)	72 (6.6)	0.985
Systolic blood pressure (mmHg)	133.0 [115.0–146.0]	133.0 [116.0–148.0]	0.906
Systolic blood pressure < 100 (mmHg)	187 (7.4)	94 (8.6)	0.214
Diastolic blood pressure (mmHg)	72.0 [61.0–80.0]	74.0 [62.0–82.0]	0.695
Mean arterial pressure (mmHg)	93.3 [80.8–101.0]	93.7 [81.5–101.8]	0.760
Heart rate (beats/min)	84.0 [73.0–96.0]	84.0 [74.0–97.5]	0.250
Respiratory rate (beats/min)	18.0 [16.0–19.0]	18.0 [16.0–18.0]	0.286
O_2_ saturation (%)	98.0 [96.0–99.0]	98.0 [97.0–99.0]	0.451
Comorbidities, *n* (%)			
Hypertension	1386 (54.7)	590 (54.3)	0.855
Diabetes mellitus	739 (29.2)	341 (31.4)	0.194
Myocardial infarction	330 (13.0)	156 (14.4)	0.305
Congestive heart failure	646 (25.5)	277 (25.5)	1.000
Prior stroke	304 (12.0)	129 (11.9)	0.960
Liver disease	282 (11.1)	106 (9.8)	0.244
Chronic kidney disease	672 (26.5)	291 (26.8)	0.902
Malignancy	341 (13.5)	137 (12.6)	0.524
Charlson comorbidity index	5.8 (3.8)	5.7 (3.9)	0.590
Laboratory data (serum), median [IQR]			
WBC (103/uL)	8.7 [6.4–11.9]	8.6 [6.4–11.8]	0.115
Neutrophil (%)	77.5 [67.9–84.7]	75.8 [66.0–83.5]	0.098
Monocyte (%)	6.3 [4.5–8.8]	6.6 [4.7–9.0]	0.237
Lymphocyte (%)	12.1 [7.3–19.6]	12.9 [8.0–20.9]	0.129
Hemoglobin (g/dL)	10.4 [8.9–12.0]	10.6 [9.0–12.1]	0.285
MCV (fL)	92.0 [87.0–96.0]	92.0 [87.0–96.0]	0.985
RDW (%)	14.9 [13.8–16.6]	14.7 [13.6–16.4]	0.015
Platelet count (10^3^/uL)	214.0 [156.0–278.0]	213.0 [160.0–276.5]	0.893
PT (s)	13.2 [11.8–17.4]	12.9 [11.7–15.8]	0.384
aPTT (s)	33.5 [28.7–37.5]	33.6 [28.6–37.7]	0.425
Sodium (mEq/L)	139.0 [136.0–141.0]	139.0 [135.5–141.0]	0.846
Potassium (mEq/L)	4.1 [3.7–4.5]	4.1 [3.7–4.5]	0.794
AST (U/L)	30.2 [19.0–55.0]	29.5 [18.3–53.0]	0.123
ALT (U/L)	22.0 [12.0–42.0]	20.7 [12.0–38.5]	0.043
Total bilirubin (mg/dL)	0.5 [0.3–0.9]	0.5 [0.3–0.9]	0.106
Glucose (mg/dL)	114.0 [94.0–149.0]	115.0 [95.0–151.5]	0.331
Blood urea nitrogen (mg/dL)	22.0 [15.0–37.0]	22.0 [14.0–35.0]	0.303
Creatinine (mg/dL)	1.0 [0.7–1.6]	1.0 [0.7–1.6]	0.746
Albumin (g/dL)	3.2 [2.9–3.5]	3.2 [2.9–3.5]	0.503
Calcium (mg/dL)	8.7 [8.2–9.2]	8.7 [8.3–9.1]	0.746
Magnesium (mg/dL)	1.9 [1.7–2.1]	1.9 [1.7–2.1]	0.630
Chloride (mEq/L)	102.0 [99.0–106.0]	103.0 [99.0–106.0]	0.575
Lactate (mmol/L)	1.7 [1.4–2.0]	1.7 [1.4–2.0]	0.340
C-reactive protein (mg/L)	51.0 [14.0–103.9]	47.6 [18.9–99.5]	0.800
pH	7.4 [7.3–7.4]	7.4 [7.3–7.4]	0.596
Bicarbonate (mmol/L)	24.0 [21.0–27.0]	24.0 [21.0–27.0]	0.340
Urinalysis, median [IQR] ^†^			
WBC (/HPF)	19.0 [6.0–57.0]	22.0 [6.0–59.0]	0.303
RBC (/HPF)	5.0 [2.0–15.0]	5.0 [2.0–16.5]	0.128
pH	6.0 [5.5–6.5]	6.0 [5.5–6.5]	0.635
Urine culture, *n* (%)			0.068
*Escherichia coli*	900 (35.5)	396 (36.4)	
*Enterococcus* sp.	365 (14.4)	152 (14.1)	
Yeast	294 (11.6)	92 (8.5)	
*Klebsiella* sp.	280 (11.0)	116 (10.7)	
*Proteus* sp.	120 (4.8)	47 (4.3)	
*Pseudomonas* sp.	113 (4.5)	50 (4.6)	
Other microorganisms	463 (18.2)	234 (21.5)	
Prediction score			
MEWS	1.6 (1.0)	1.6 (1.1)	0.297
NEWS	1.4 (1.6)	1.4 (1.7)	0.630
REMS	5.8 (2.2)	5.8 (2.3)	0.809
Critical outcomes, *n* (%)	836 (33.0)	358 (32.9)	1.000
ICU admission	766 (30.2)	329 (30.3)	1.000
In-hospital mortality	153 (6.0)	67 (6.2)	0.942

IQR: interquartile range; WBC: white blood cell; MCV: mean corpuscular volume; RDW: red cell distribution width; PT: prothrombin time; aPTT: activated partial thromboplastin time; AST: aspartate aminotransferase; ALT: alanine aminotransferase; RBC: red blood cell; ICU: intensive care unit; MEWS: modified early warning score; NEWS: national early warning score; REMS: rapid emergency medicine score. ^†^ Defined as body temperature > 38 °C.

**Table 2:** Comparison of selected variables from stepwise approach in patients with or without critical outcomes.

	Without Critical Outcomes (*n* = 1699)	With Critical Outcomes (*n* = 836)	*p* Value
Emergency severity index, *n* (%)			<0.001
Level 1	153 (9.0)	299 (35.8)	
Level 2	874 (51.4)	433 (51.8)	
Level 3	664 (39.1)	104 (12.4)	
Level 4	8 (0.5)	0 (0.0)	
Arrival by ambulance, *n* (%)	1118 (65.8)	571 (68.3)	0.227
Mean arterial pressure, median [IQR]	91.7 [81.3–102.7]	95.7 [80.0–98.1]	0.289
WBC (10^3^/uL), median [IQR]	8.2 [5.9–10.9]	10.2 [7.5–14.1]	<0.001
Hemoglobin (g/dL), median [IQR]	10.4 [8.9–11.9]	10.5 [8.8–12.1]	0.59
MCV (fL), median [IQR]	92.0 [87.0–96.0]	92.0 [88.0–97.0]	0.013
Platelet count (10^3^/uL), median [IQR]	218.0 [158.0–285.5]	205.0 [150.0–263.2]	<0.001
aPTT (s), median [IQR]	35.0 [29.6–37.5]	31.3 [27.7–37.6]	<0.001
Sodium (mEq/L), median [IQR]	139.0 [136.0–141.0]	139.0 [135.0–142.0]	0.479
Blood urea nitrogen (mg/dL), median [IQR]	21.0 [14.0–34.0]	25.0 [16.0–43.0]	<0.001
Creatinine (mg/dL), median [IQR]	1.0 [0.7–1.6]	1.1 [0.8–1.7]	0.059
AST (U/L), median [IQR]	28.0 [17.0–49.9]	35.8 [22.0–68.1]	<0.001
ALT (U/L), median [IQR]	20.0 [11.0–38.0]	25.6 [15.0–51.0]	<0.001
Calcium (mg/dL), median [IQR]	8.8 [8.4–9.2]	8.6 [7.9–9.1]	<0.001
Magnesium (mg/dL), median [IQR]	1.9 [1.8–2.1]	1.9 [1.7–2.1]	0.005
Chloride (mEq/L), median [IQR]	102.0 [99.0–105.0]	103.0 [99.0–107.0]	<0.001
Glucose (mg/dL), median [IQR]	108.0 [92.0–140.0]	125.0 [102.0–166.0]	<0.001
Albumin (g/dL), median [IQR]	3.3 [3.0–3.5]	3.1 [2.7–3.4]	<0.001
Lactate (mmol/L), median [IQR]	1.7 [1.4–1.9]	1.8 [1.4–2.3]	<0.001
Bicarbonate (mmol/L), median [IQR]	25.0 [22.0–27.0]	23.0 [19.0–26.0]	<0.001

IQR: interquartile range; WBC: white blood cell; MCV: mean corpuscular volume; aPTT: activated partial thromboplastin time; AST: aspartate aminotransferase; ALT: alanine aminotransferase.

**Table 3:** Performance of four models for predicting critical outcomes in patients with UTI.

Models	AUC (95% CI)	Accuracy	No-Information Rate	Balanced Accuracy	Kappa	Precision	F1 Score	Sensitivity	Specificity
XGBoost	0.833 (0.806–0.857)	0.691	0.671	0.739	0.406	0.518	0.652	0.880	0.598
RF	0.814 (0.785–0.839)	0.557	0.671	0.656	0.237	0.237	0.237	0.947	0.365
SVM	0.799 (0.771–0.826)	0.650	0.671	0.700	0.335	0.482	0.614	0.844	0.556
GEE LR	0.793 (0.764–0.820)	0.615	0.671	0.676	0.288	0.455	0.594	0.858	0.495

## Data Availability

The datasets presented in this study are accessible through the MIMIC-IV-ED database at https://physionet.org/content/mimic-iv-ed/2.2/, accessed on 1 November 2023. While the datasets are de-identified, restrictions have been placed on data sharing due to the sensitive nature of the information they contain. Researchers seeking access must first sign the relevant convention. Additionally, interested parties must meet specific criteria: they must be credentialed users of https://physionet.org/ (accessed on 1 November 2023), complete required training, and sign the data use agreement for the project. Furthermore, all the code utilized in this project is openly available on GitHub at https://github.com/mournepic/UTI-predictive-model.git (accessed on 1 November 2023).

## Supplementary Materials

The following supporting information can be downloaded at: https://www.mdpi.com/article/10.3390/diagnostics14171974/s1, Table S1: The summary descriptions of the selected machine learning algorithms; Table S2: Confusion matrix of each model for critical outcomes in patients with UTI; Table S3: Variance inflation factor values in the logistic regression model; Table S4: Performance of the XGBoost model for predicting critical outcomes in patients with UTI; Table S5: Performance of four models for predicting in-hospital mortality in patients with UTI; Figure S1: Frequency of missing data; Figure S2: Comparison of AUC of four models to traditional prediction tools for predicting in-hospital mortality in patients with UTI; Figure S3: Relationship between ESI, bicarbonate, magnesium, albumin, AST, ALT, and predicted outcomes; Figure S4: Relationship between hemoglobin, MAP, MCV, calcium, chloride, creatinine, and predicted outcomes; Figure S5: Relationship between glucose, sodium, aPTT, BUN, platelet count, WBCs, and predicted outcomes; Figure S6: Relationship between lactate, ambulance, and predicted outcomes; Figure S7: Break-down profile.
