# Supplementary material for: Interpretable Machine Learning Models for Predicting Critical Outcomes in Patients with Suspected Urinary Tract Infection with Positive Urine Culture
Source: Diagnostics (Basel). 2024 Sep 6;14(17):1974. doi: 10.3390/diagnostics14171974 (PMC11394224; doi:10.3390/diagnostics14171974)

Table S1. The summary descriptions of the selected machine learning algorithms

Algorithm	Description
XGBoost	eXtreme Gradient Boosting (XGBoost) is a decision-tree-based ensemble algorithm that uses boosting techniques to improve the accuracy of predictions by focusing on the misclassified observations in each iteration. XGBoost is known for its high performance and efficiency, making it suitable for a wide range of classification and regression tasks. It handles multicollinearity effectively through its tree-based approach, which automatically selects the most important features, reducing the impact of correlated variables.
Random Forest	Random Forest (RF) forms an ensemble of bagged decision tree models. Each tree is built using a random subset of features and data samples, which helps to reduce overfitting and variance. The final prediction is obtained by aggregating the outputs of all trees, typically by taking the mode for classification tasks or the mean for regression tasks. Random Forest naturally mitigates multicollinearity by randomly selecting features for each tree, which lessens the dependency on correlated variables.
Support Vector Machine	A Support Vector Machine (SVM) constructs hyperplanes in a high- or infinite-dimensional space, used for classification,

	regression, or outlier detection. SVM is particularly effective in cases where the data are not linearly separable in its original space, as it uses kernel functions to map data into higher-dimensional spaces, facilitating better separation and classification.

Table S2. Confusion matrix of each model for critical outcomes in patients with UTI

XGBoost model	Predicted negative	Predicted positive
True negative	436	293
True positive	43	315
RF model	Predicted negative	Predicted positive
True negative	266	463
True positive	19	339
SVM model	Predicted negative	Predicted positive
True negative	405	324
True positive	56	302
GEE LR model	Predicted negative	Predicted positive
True negative	361	368
True positive	51	307

UTI: urinary tract infection; RF: random forest; SVM: support vector machine; GEE LR: generalized estimating equation logistic regression model

Table S3. Variance inflation factor values in the logistic regression model

Variable	Variance inflation factor
Emergency severity index	1.03556
Arrival by ambulance	1.023625
Mean arterial pressure	1.137846
WBC	1.220317
Hemoglobin	1.452459
MCV	1.094532
Platelet count	1.256914
aPTT	1.039162
Sodium	4.485324
Blood urea nitrogen	2.596427
Creatinine	2.346261
AST	2.775899

ALT	2.623097
Calcium	1.486976
Magnesium	1.313468
Chloride	5.270422
Glucose	1.082458
Albumin	1.745744
Lactate	1.190864
Bicarbonate	2.299777

WBC: white blood cell; MCV: mean corpuscular volume; aPTT: activated partial thromboplastin time; AST: aspartate aminotransferase; ALT: alanine aminotransferase.

Table S4. Performance of the XGBoost model for predicting critical outcomes in patients with UTI

Subgroups	AUC (95% CI)	Accuracy	No-Information Rate	Balanced Accuracy	Kappa	Precision	F1 Score	Sensitivity	Specificity
Patients with fever[†]	0.758 (0.631-0.861)	0.639	0.708	0.703	0.317	0.439	0.581	0.857	0.549
Patients with SBP≥100 mmHg	0.825 (0.795-0.853)	0.681	0.688	0.731	0.383	0.494	0.628	0.865	0.597
Patients with SBP<100 mmHg	0.870 (0.794-0.931)	0.798	0.489	0.794	0.592	0.723	0.832	0.979	0.609

CI: confidence interval; UTI: urinary tract infection; AUC: area under the curve; SBP: systolic blood pressure.

† Defined as body temperature > 38 °C

Table S5. Performance of four models for predicting in-hospital mortality in patients with UTI

Models	AUC (95% CI)	Accuracy	No-Information Rate	Balanced Accuracy	Kappa	Precision	F1 Score	Sensitivity	Specificity
XGBoost	0.775 (0.727-0.821)	0.909	0.938	0.589	0.184	0.242	0.233	0.224	0.954
RF	0.746 (0.677-0.808)	0.939	0.938	0.514	0.052	0.667	0.057	0.030	0.999
SVM	0.715 (0.637-0.784)	0.916	0.938	0.530	0.077	0.167	0.117	0.090	0.971
GEE LR	0.740 (0.667-0.807)	0.916	0.938	0.593	0.204	0.278	0.248	0.224	0.962

CI: confidence interval; UTI: urinary tract infection; AUC: area under the curve; RF: random forest; SVM: support vector machine; GEE LR: generalized estimating equation logistic regression model

Figure S1. Frequency of missing data. ALT: alanine aminotransferase; AST: aspartate aminotransferase; aPTT: activated partial thromboplastin time; PT: prothrombin time; RDW: red cell distribution width; MCV: mean corpuscular volume.

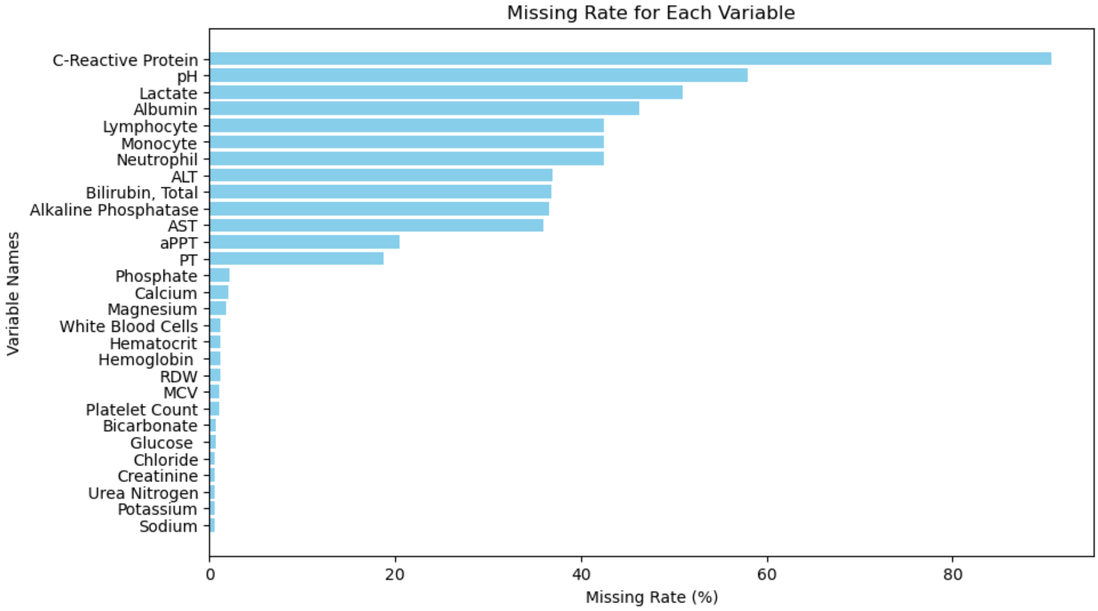

Figure S2. Comparison of AUC of four models to traditional prediction tools for predicting in-hospital mortality in patients with UTI. AUC: area under the curve; UTI: urinary tract infection.

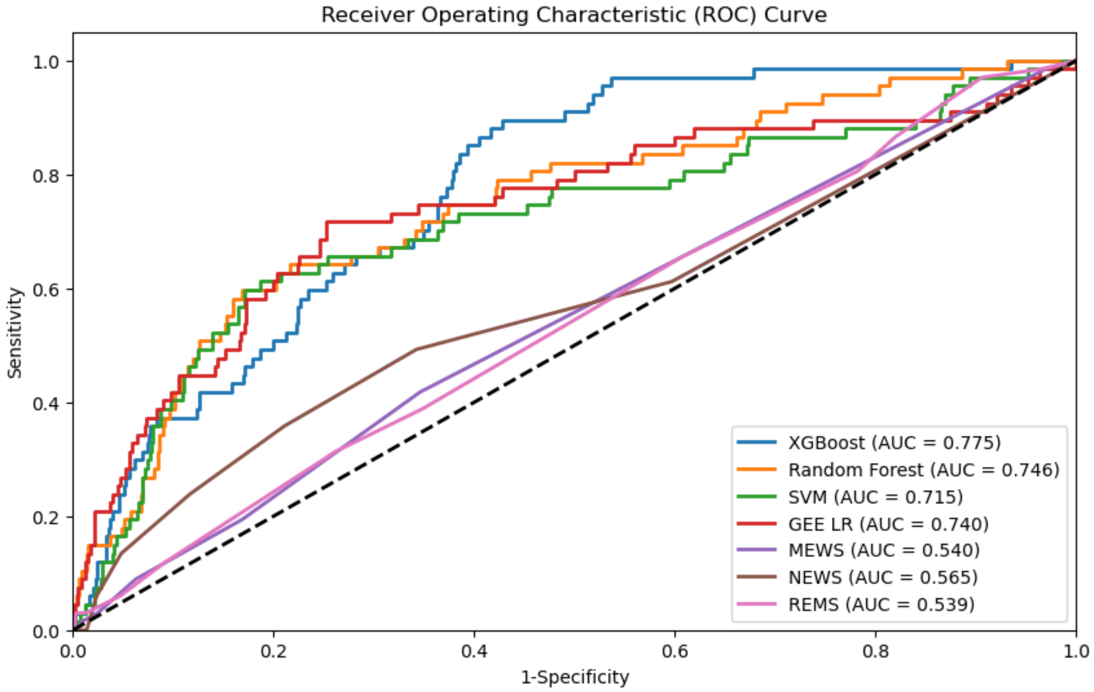

Figure S3. Relationship between ESI, bicarbonate, magnesium, albumin, AST, ALT, and predicted outcomes. ESI: emergency severity index; AST: aspartate aminotransferase; ALT: alanine aminotransferase.

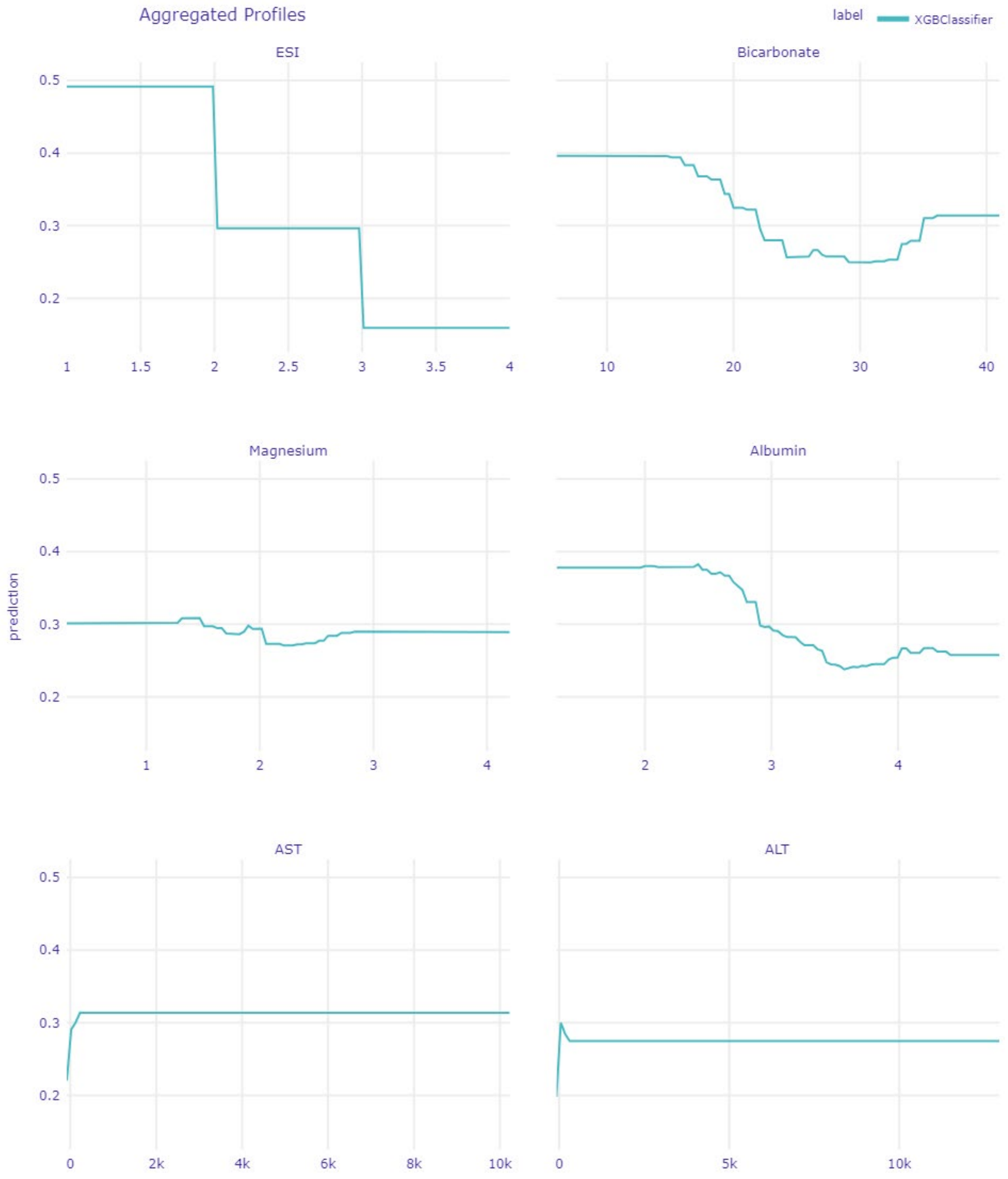

This figure was generated using the DALEX package. It illustrates the relationship between ESI, bicarbonate, magnesium, albumin, AST, ALT, and predicted outcomes. Higher levels of AST and ALT, as well as lower levels of ESI, were found to be linked to a higher risk of critical outcomes. On the other hand, albumin and bicarbonate exhibited a U-shaped curve, indicating that both low and high levels of these variables were associated with an increased risk of critical outcomes.

Figure S4. Relationship between hemoglobin, MAP, MCV, calcium, chloride, creatinine, and predicted outcomes. MAP: mean arterial pressure; MCV: mean corpuscular volume.

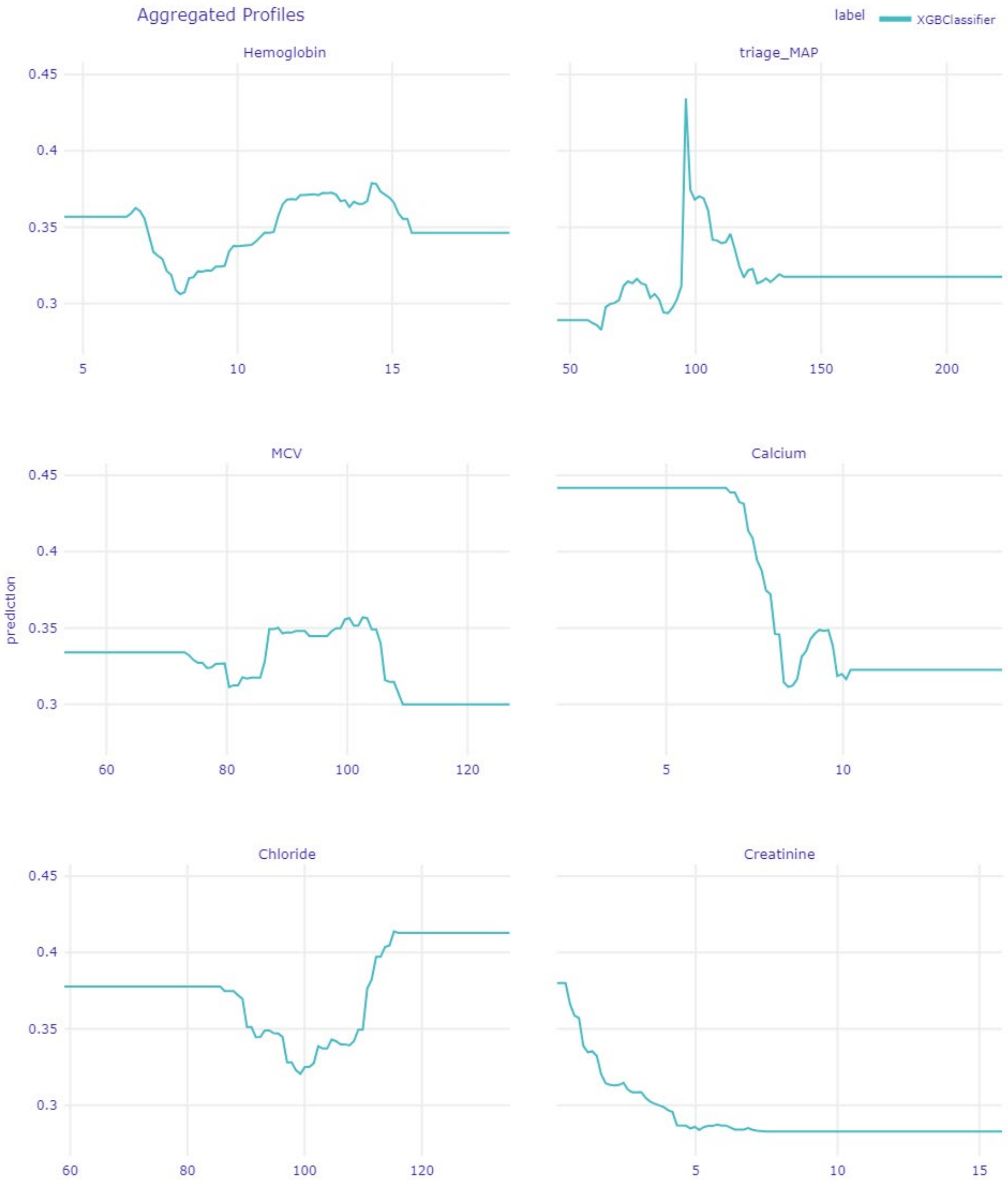

This figure was generated using the DALEX package. It illustrates the relationship between hemoglobin, MAP, MCV, calcium, chloride, creatinine, and predicted outcomes. Lower levels of calcium were found to be linked to a higher risk of critical outcomes. On the other hand, chloride exhibited a U-shaped curve, indicating that both low and high levels of these variables were associated with an increased risk of critical outcomes.

Figure S5. Relationship between glucose, sodium, aPTT, BUN, platelet count, WBCs, and predicted outcomes. aPTT: activated partial thromboplastin time; BUN: blood urea nitrogen; WBCs: white blood cells.

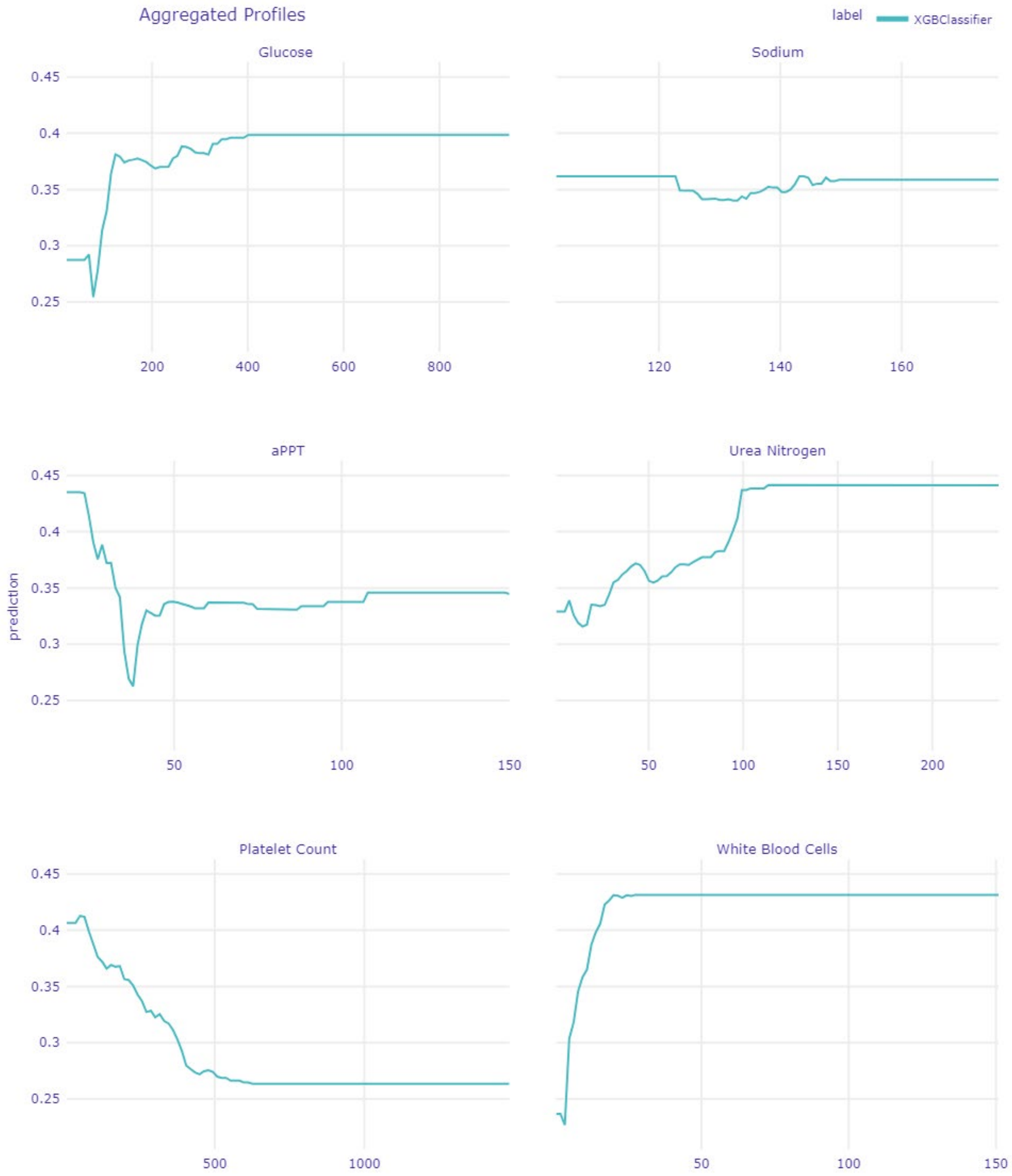

This figure was generated using the DALEX package. It illustrates the relationship between glucose, sodium, aPTT, BUN, platelet count, WBCs, and predicted outcomes. Higher levels of glucose, BUN, and WBCs, as well as lower levels of platelet count, were found to be linked to a higher risk of critical outcomes. On the other hand, aPTT exhibited a U-shaped curve, indicating that both low and high levels of these variables were associated with an increased risk of critical outcomes.

Figure S6. Relationship between lactate, ambulance, and predicted outcomes.

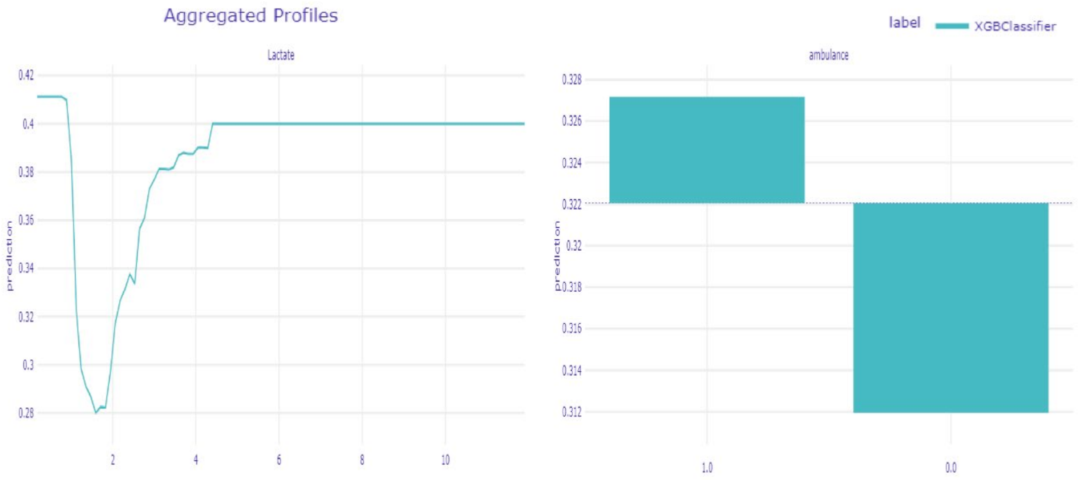

This figure was generated using the DALEX package. It illustrates the relationship between lactate, ambulance, and predicted outcomes. Arrival by ambulance was found to be linked to a higher risk of critical outcomes. Lactate exhibited a U-shaped curve, indicating that both low and high levels of these variables were associated with an increased risk of critical outcomes.

Figure S7. Break-down profile. This patient, a 49-year-old male, presented with flank pain and was brought in by an ambulance. At the initial ED presentation, his triage MAP was 92.66 mmHg. Laboratory data revealed a hemoglobin level of 11.3 g/dL, hematocrit at 43.3%, MCV at 80 fL, platelet count at 237 K/uL, and white blood cell count at 7.8 K/uL. His albumin level stood at 3.43 g/dL, serum creatinine at 1.3 mg/dL, aPTT at 37 s, glucose at 120 mg/dL, sodium at 141 mmol/L, urea nitrogen at 16 mg/dL, and total bilirubin at 0.7 mg/dL. ALT and AST levels were 50 U/L and 51 U/L, respectively. Additional markers indicated bicarbonate at 22 mmol/L, magnesium at 1.5 mmol/L, calcium at 9.3 mg/dL, chloride at 107 mmol/L, and lactate at 1.69 mmol/L. The XGBoost model predicted a 3.1% risk of critical outcomes based on the patient's initial ED presentation clinical characteristics, with glucose, bicarbonate, ALT, hemoglobin, and calcium identified as the top five contributors to the increased risk of critical outcomes, while aPTT notably reduced the model's prediction the most. The predicted outcome was that the patient would not experience critical outcomes, which was consistent with the actual outcome (true negative).

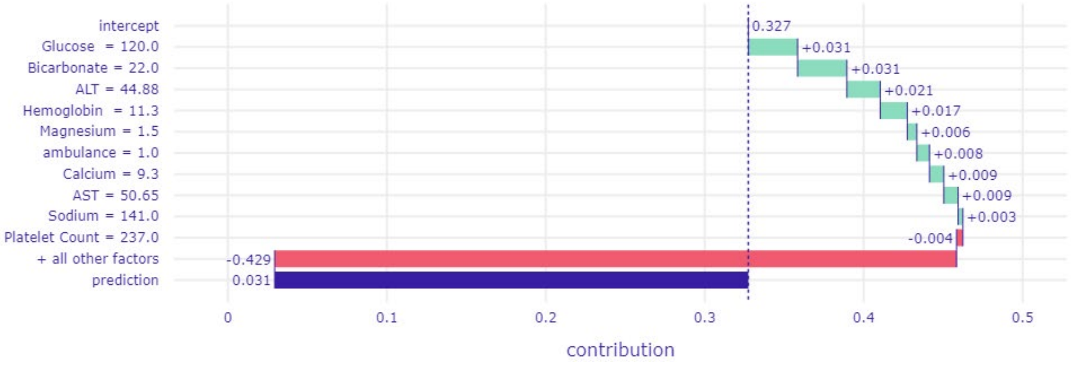

Supplement: Supplementary file 1 [file diagnostics-14-01974-s001.zip › diagnostics-3110095-supplementary.pdf]
